# Higher risk of neoplastic progression of Barrett’s esophagus in patients with systemic sclerosis

**DOI:** 10.1093/gastro/goaa096

**Published:** 2021-01-30

**Authors:** Mythri Anilkumar, Motasem Alkhayyat, Udhayvir S Grewal, Madhusudhan R Sanaka, Prashanthi N Thota

**Affiliations:** 1 Center of Excellence for Barrett’s Esophagus, Digestive Disease Institute, Cleveland Clinic, Cleveland, OH, USA; 2 Department of Internal Medicine, Louisiana State University Health Shreveport, Shreveport, LA, USA

Systemic sclerosis (SSc) is a multisystem connective-tissue disorder of unknown etiology affecting various internal organs and skin. Esophageal involvement is present in ≤70%–90% of the patients and is manifested by severe gastroesophageal-reflux disease and dysmotility. Although Barrett’s esophagus (BE) has been reported in patients with SSc [1, 2], the risk of neoplastic progression has not been reported before in the USA. Therefore, we aimed to study the prevalence of BE in SSc and incidence of neoplastic progression.

After obtaining approval from our institutional review board, we identified patients >18 years of age with a confirmed diagnosis of BE and a prior history of SSc from 1 January 1997 to 31 December 2017. Data including demographics, clinical features, laboratory findings, and endoscopic and histological findings were abstracted from the medical records. Fifty-six patients with both BE and SSc were identified in our center. The cohort was predominantly female (41/56, 73.2%) and white (51/56, 91.1%) with a mean age of 65.2 ± 12.0 years and body mass index of 24.9 ± 6.3 kg/m^2^. Heartburn, regurgitation, chest pain, dysphagia, and weight loss were reported in 25%, 50%, 66.1%, 42.9%, and 62.5% of the patients, respectively. Tobacco use was reported in 42.9% (24/56) while alcohol use was reported in 17.9% (10/56) of the patients. Depending on the extent of skin involvement, two predominant subsets of SSc exist: diffuse and limited. In the diffuse type, the skin involvement is proximal to the elbows or knees, whereas, in the limited type, skin involvement is restricted to the limbs distal to the elbows or knees, with or without face and neck involvement. The limited type of SSc was more common (46/56, 82.1%) than diffuse SSc. Nearly half the cohort (24/56, 42.9%) had interstitial lung disease and most patients reported Raynaud’s phenomenon (52/56, 92.9%). Autoantibody test results were as follows: 78.8% (26/33) were positive for antinuclear antibodies, 86.2% (25/29) were positive for anti-centromere antibodies, and 23.7% (9/38) were positive for anti-SSc antibodies. Endoscopy revealed reflux esophagitis in 20 (35.7%) patients, esophageal stricture in 7 (12.5%) patients, and dilated esophageal lumen in 9 (16.1%) patients. The mean BE length was 3.6 ± 2.4 cm. Hiatal hernia was observed in 34 (60.7%) patients, with a mean length of 1.8 ± 2.0 cm.

On index endoscopy, 51 (91.1%) patients had non-dysplastic BE (NDBE), 1 (1.8%) patient had indefinite for dysplasia (IND), 1 (1.8%) patient had low-grade dysplasia (LGD), and 3 patients (5.4%) had invasive esophageal adenocarcinoma (EAC). Among the 51 patients, 24 were followed for >1 year. During a mean follow-up of 57.2 ± 9.7 months, two females developed high-grade dysplasia (HGD) and one male developed intramucosal cancer (IMC), leading to an annual HGD/EAC progression rate of 2.62%. In the remaining patients, 18 had persistent NDBE, 1 developed IND, 1 developed LGD, and 1 regressed to no metaplasia.

Even though the prevalence of BE in SSc patients varies between 16% and 37% [1, 2], routine screening for BE is not widely performed. We observed that SSc patients with BE are at high risk of neoplastic progression, with an incidence rate of HGD/EAC of 2.62% per year. This is significantly higher than the 0.48% per year reported in general NDBE patients [3] and 0.1% annual risk reported in women [4]. It is worthwhile to note that our patients were predominantly female non-smokers, which is markedly different from the classic BE patients, who are white males and obese. There is a paucity of literature on BE and EAC in SSc patients. In a European study of 36 BE patients with SSc, one developed HGD and none developed EAC during 111 patient-years of follow-up [5]. A few studies reported on development of esophageal cancer in SSc patients without any mention of BE. Derk *et al.* [6] reported 7 cases of esophageal cancer in a cohort of 769 SSc patients with an incidence rate of 0.18% per year, which translates into a 16-times increased risk compared to an age-matched population. In an Australian cohort, one esophageal cancer developed among 363 SSc women followed for 6.1 ± 2.8 years [7]. In the Michigan SSc registry, 1 in 538 SSc patients developed esophageal cancer over a total follow-up of 5,766.14 person-years [8]. In conclusion, we observed an increased risk of progression in SSc patients with BE, which highlights the need for more rigorous surveillance. Prospective studies are needed to confirm this increased risk and possible early intervention.

## Authors’ contributions

M.An., M.Al., and U.G.: acquisition, analysis and interpretation of data, drafting of the manuscript; M.R.S.: critical revision of the manuscript for important intellectual content; P.N.T.: study concept and design, critical revision of the manuscript for important intellectual content.

## Funding

P.N.T. is supported by NIH grants [U54CA163060 and P50CA150964]. All other authors disclose no funding support.

## Conflicts of interest

None declared.
